# Indoor Pedestrian Localization Using iBeacon and Improved Kalman Filter

**DOI:** 10.3390/s18061722

**Published:** 2018-05-26

**Authors:** Kwangjae Sung, Dong Kyu ‘Roy’ Lee, Hwangnam Kim

**Affiliations:** School of Electrical Engineering, Korea University, Seoul 02841, Korea; kjsung80@korea.ac.kr (K.S.); roylee6315@korea.ac.kr (D.K.R.L)

**Keywords:** sensor fusion, Kalman filtering, particle filtering, indoor positioning, dead reckoning, received signal strength (RSS) fingerprinting, Bluetooth beacon, Bluetooth Low Energy

## Abstract

The reliable and accurate indoor pedestrian positioning is one of the biggest challenges for location-based systems and applications. Most pedestrian positioning systems have drift error and large bias due to low-cost inertial sensors and random motions of human being, as well as unpredictable and time-varying radio-frequency (RF) signals used for position determination. To solve this problem, many indoor positioning approaches that integrate the user’s motion estimated by dead reckoning (DR) method and the location data obtained by RSS fingerprinting through Bayesian filter, such as the Kalman filter (KF), unscented Kalman filter (UKF), and particle filter (PF), have recently been proposed to achieve higher positioning accuracy in indoor environments. Among Bayesian filtering methods, PF is the most popular integrating approach and can provide the best localization performance. However, since PF uses a large number of particles for the high performance, it can lead to considerable computational cost. This paper presents an indoor positioning system implemented on a smartphone, which uses simple dead reckoning (DR), RSS fingerprinting using iBeacon and machine learning scheme, and improved KF. The core of the system is the enhanced KF called a sigma-point Kalman particle filter (SKPF), which localize the user leveraging both the unscented transform of UKF and the weighting method of PF. The SKPF algorithm proposed in this study is used to provide the enhanced positioning accuracy by fusing positional data obtained from both DR and fingerprinting with uncertainty. The SKPF algorithm can achieve better positioning accuracy than KF and UKF and comparable performance compared to PF, and it can provide higher computational efficiency compared with PF. iBeacon in our positioning system is used for energy-efficient localization and RSS fingerprinting. We aim to design the localization scheme that can realize the high positioning accuracy, computational efficiency, and energy efficiency through the SKPF and iBeacon indoors. Empirical experiments in real environments show that the use of the SKPF algorithm and iBeacon in our indoor localization scheme can achieve very satisfactory performance in terms of localization accuracy, computational cost, and energy efficiency.

## 1. Introduction

The Global Positioning System (GPS) is commonly used for navigation in outdoor environments. However, it is not available for indoor positioning due to the obstruction of signals. As a result, various indoor navigation approaches have been extensively studied to achieve reliable and high accuracy positioning for a person or a device indoors. Researchers have proposed many solutions using radio beacons, inertial sensors, ultrasound, vision, etc. Most indoor positioning systems are classified into two types: non-infrastructure-based (e.g., DR scheme) and infrastructure-based (e.g., RSS fingerprinting).

DR-based method, also known as an inertial navigation system (INS), is a self-contained localization system that relies on inertial sensors such as accelerometer, gyroscope, and magnetometer without the assistance of the GPS and infrastructure [1,2]. Pedestrian navigation system (PNS) as an instance of DR technique estimates the location of the user by measuring the traveled distance and direction from a known location using the motion sensors. However, low cost sensors may have drift error and large bias. In addition, positioning errors in DR can be caused by an oscillation of user body during the walking.

RSS fingerprinting is a kind of the localization method using the received signal strength (RSS) from the radio beacons such as WiFi access points (APs) [3,4], Bluetooth devices [5], and cellular telephone towers [6]. This approach consists of two phases: offline and online. During the offline phase, RSS fingerprints are recorded at known locations in order to build a fingerprint map (database). Next, in the online step, the measured RSS values are compared to the map for locating the user. Although creating the fingerprint map through site survey requires considerable cost and time, since RSS fingerprints reflect the spatial radio characteristics about a given location well, the fingerprinting-based approaches have been widely used to estimate the location of the user. However, since the radio-frequency (RF) signals can vary over time and space because of obstacles and multipath fading, it may degrade the performance of the positioning using RSS fingerprints.

To achieve better indoor localization results, many smartphone-based localization approaches that integrate both DR and fingerprinting method through Bayesian filter, such as the Kalman filter (KF), unscented Kalman filter (UKF), and particle filter (PF), have recently been proposed [7,8,9]. Among Bayesian filters, PF is the most popular integrating scheme and can provide the best localization performance. For the localization scheme based on PF, the location of the user is predicted by user motion measured from inertial sensors and is corrected by positional information obtained from WiFi fingerprints [10,11,12]. He et al. [4] is a method that combines step counter and WiFi fingerprints to optimize location estimation problem, in which user’s mobility and wireless signals are jointly employed through a specialized particle filter. It learns parameters during step mode of each user (the relationship between stride length and step frequency), calibrates RSS readings of heterogeneous devices, and simultaneously infers positions of walking users by solving a convex optimization problem. Shu et al. [13] used the gradient of WiFi RSS readings to deal with the time-varying wireless signal strength and biased RSS values across devices along with the changing transmission power of WiFi routers. It first builds a RSS gradient-based fingerprint map (Gmap) by comparing absolute RSSI measurements at nearby locations, and then carries out an online extended particle filter (EPF) to estimate the user’s location. In EPF algorithm, the user’s location is predicted by detected mobility and is updated by comparing results between RSS readings and Gmap. However, the existing positioning schemes cannot be suitable for LBS applications that require real-time positional information, because of high computational cost of PF. The excessive sample (particle) sizes of PF required for the high positioning performance can lead to a considerable amount of computational time compared with KF and UKF.

Recently, iBeacon introduced as a new kind of Bluetooth transmitter is Apple’s implementation of Bluetooth low-energy (BLE) wireless technology to provide location-based information and services to smartphones and other mobile devices [7]. Since iBeacon can help smartphones determine their physical position or context as a new class of low-powered and low-cost transmitters, it can offer a good chance to enhance the existing localization approaches [14,15].

In this paper, we present a localization system that leverages simple DR, RSS fingerprinting using iBeacon and machine learning scheme, and enhanced KF. Using the DR method, the position of the user is predicted by the sensory data (acceleration and heading) of the smartphone. Instead of GPS, the positional measurement of the user can be obtained from the RSS fingerprinting approach using energy-efficient iBeacon and machine learning approaches, such as ANN (artificial neural network), KNN (k-nearest neighbors algorithm), NBC (naive Bayes classifier), and SVM (support vector machine) as in [16]. However, there can be still errors (uncertainties) in positional information obtained by both the DR method and RSS fingerprinting.

The core of our localization system is the enhanced KF called a sigma-point Kalman particle filter (SKPF), which estimates the position of the user using both the unscented transformation of UKF and the weighting method of PF. The SKPF algorithm proposed in this study is used to provide the enhanced positioning accuracy by integrating noisy positional information estimated by DR method and the location data obtained by RSS fingerprinting with uncertainty. The SKPF algorithm can achieve better positioning accuracy than KF and UKF and comparable performance compared to PF, and it can provide higher computational efficiency compared with PF. We aim to design the localization scheme that can realize the high positioning accuracy, computational efficiency, and energy efficiency through the SKPF and iBeacon indoors. Empirical results in a building show that the use of the SKPF in our indoor localization system can achieve very satisfactory performance in aspect of positioning accuracy and computational cost compared with KF, UKF, and PF. It is also shown in the test results that the positioning system using iBeacon receiver for the RSS fingerprinting can provide more energy-efficient localization than using WiFi module.

The rest of this paper is organized as follows. Section 2 describes a summary of related work. Next, Section 3 describes the overall system architecture. Each component of our positioning system is addressed in Section 4. Section 5 and Section 6 show the experimental testbed and results, respectively. Finally, Section 7 provides conclusions of this paper.

## 2. Related Work

Most non-infrastructure-based localization techniques adopt dead reckoning (DR) as the basic scheme for positioning. DR-based methods depend on an inertial measurement unit (IMU) that contains accelerometer, gyroscope, and magnetometer to calculate the position, orientation, and velocity of the object without the help of the infrastructure [17]. Pedestrian navigation system (PNS) is an instance of the DR approach. In PNS, a pedestrian model that contains the step detection, stride length calculation, and heading inference is a key component. The step of the user is detected by counting the number of the peak value for acceleration measured by accelerometer [18]. The step length of the user is estimated by analyzing human walking patterns using walking speed and frequency [19]. The gyroscope and magnetometer (digital compass) are exploited to determine the heading angle of the pedestrian.

However, low-cost MEMS IMUs may be prone to drift error and large bias. Furthermore, the irrelevant motion of the pedestrian can degrade the performance of the positioning system. Consequently, to rectify the pedestrian location, many sophisticated and complex DR approaches have been studied [20,21]. Compared to these works, our DR method can locate the pedestrian using only the heading information and the peak value of the acceleration without complex process.

The fingerprinting-based techniques require no prior knowledge about infrastructure locations and no propagation model. The main idea is to create a fingerprint map (database) by fingerprinting the surrounding features at every position in the area of interest and then to estimate the associated position by mapping the measured feature against the fingerprint map. RADAR [22] is an early fingerprinting method. Varshavsky et al. [23] and LANDMARC [24] utilizes GSM signals from cellular radio towers and active RFID for indoor positioning, respectively. PlaceLab [6] demonstrates the use of radio beacons, such as WiFi APs, cellular radio towers, and Bluetooth devices, for device positioning in the wild. Likewise, most fingerprinting approaches use a variety of ambient features and need dense infrastructure for higher positioning accuracy [25,26]. Recently, some fingerprinting methods focus on employing the received signal strength (RSS) of iBeacon modules as a surrounding feature [5,27]. Due to the usage of iBeacon based on BLE, they are more energy-efficient compared with the existing Bluetooth and WiFi modules. However, since the radio-frequency (RF) signals from radio beacons can vary over time and space due to obstructions and multipath fading, it can degrade the performance of the positioning using RSS fingerprints.

In the positioning and tracking system, the Bayesian filter is employed to fuse sensory data from all different sources to gain better positioning accuracy. Among Bayesian filtering methods, the Kalman filter (KF) and its variants, such as unscented Kalman filter (UKF), have been widely used for the navigation systems, since they are efficient in terms of the computational cost while providing a high accuracy in localization [9,28]. Recently, many indoor positioning approaches that fuse the user’s motion estimated by DR method and the location data obtained by RSS fingerprinting through PF have been proposed to achieve higher positioning accuracy than KF and its variants in indoor environments [12,29]. However, since PF requires a large number of particles (samples) for high positioning performance, it can result in substantial computational cost compared with KF and its variants.

In this paper, we propose an enhanced KF called a sigma-point Kalman particle filter (SKPF). By leveraging the unscented transformation of UKF and the weighting method of PF, the SKPF algorithm can achieve better positioning performance than KF and UKF and competitive performance compared to PF, and it can provide higher computational efficiency compared with PF. The SKPF algorithm in this study is used to achieve the enhanced positioning performance by integrating noisy positional information estimated by DR method and the location data obtained by RSS fingerprinting with uncertainty.

## 3. System Architecture

**Figure 1:**
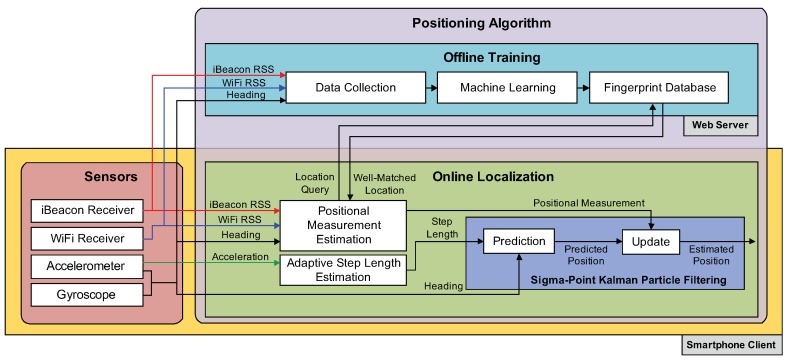
Overall architecture of our positioning system.

Figure 1 represents the overall architecture of our indoor positioning system implemented on a smartphone (iPhone5S) and web server. The smartphone client is used to measure sensing data from its built-in sensors and localize the user. The web server is employed to execute the machine learning used for positioning and receive location queries from the smartphone client. Our positioning system can be classified into a sensor part and a positioning algorithm part.

The sensor part consists of RF receivers and a group of inertial sensors on the smartphone. The RF receivers include WiFi and iBeacon modules, which provide the positioning algorithm with RSS readings measured from WiFi access points and iBeacons, respectively. The inertial sensors include the accelerometer and gyroscope on the smartphone, which measure three-axis acceleration and rotation rate of the phone. The sensory data are used for the machine learning and location estimation in the positioning algorithm.

The positioning algorithm can be broken down into two main component parts: offline training and online localization. In the offline phase, the RSS fingerprints obtained from WiFi APs and iBeacons using the smartphone and the heading information of the user gained by the accelerometer and the gyroscope on the phone are collected at selected locations in the area of interest and are sent to the server. Then, a machine learning method, such as ANN, KNN, NBC, and SVM, on the server side converts the collected fingerprints (RSS readings and user’s heading) into vectors (fingerprint database).

The online localization contains three phases: step length determination, positional measurement estimation, and sigma-point Kalman particle filtering (SKPF). The SKPF algorithm is composed of a prediction (dead reckoning) phase and an update phase, and it estimates the current position of the user with the smartphone from the two-phase process using the pedestrian model in Section 4.3.

For every step of the user, the prediction phase predicts the user’s location using both the step length (displacement) calculated according to the magnitude of the acceleration measured by the accelerometer and the heading angle obtained by the accelerometer and the gyroscope. The inference of the adaptive step length and heading is discussed further in Section 4.1.

Due to the unavailability of GPS in indoor environments, the positional measurement used to correct the predicted position in the update phase of SKPF is obtained by the fingerprint database constructed using the machine learning approach implemented on the server using during the offline phase. When the smartphone client sends a location query with the information about the observed RSS value and user direction to the server, the machine learning method on the server infers the user’s location that best matches the observed information via fingerprint database and sends back the estimated position of the user to the smartphone immediately. The estimate of the positional measurement using the machine learning is addressed in Section 4.2. During the update phase, the position of the user is estimated by integrating both the positional information obtained from prediction phase and the positional measurement determined by the machine learning. The integrating approach based on SKPF is addressed in more detail in Section 4.4.

## 4. Positioning Algorithm

The following sections describe the components of the positioning algorithm used in this paper.

### 4.1. Displacement Estimation and Heading Determination

In this section, we derive a relationship between the step length and acceleration magnitude for adaptive displacement estimation (Section 4.1.1) and deal with the determination of user heading (Section 4.1.2). The relationship and heading information are used in the prediction (dead reckoning) step of SKPF.

#### 4.1.1. Displacement Estimation

**Figure 2:**
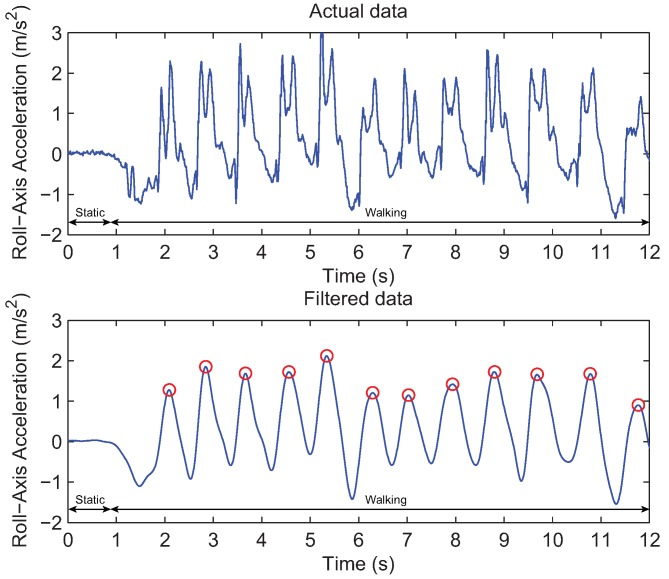
Example of the step detection using accelerometer readings. (**Top**) Raw acceleration values measured during the walking. (**Bottom**) Smoothed acceleration magnitudes using 10th-order Butterworth low pass filter. Peaks represented by red circles correspond to the user steps.

The measurements obtained from the three-axis accelerometer represent periodic patterns for each of its axes in human walking. Figure 2 (top) indicates the magnitude of roll-axis acceleration data when the user is stopping and then starts walking. Compared to the static state of the user, the variation in acceleration values during the walking has a regular pattern. For this reason, the accelerometer has been widely used to detect the user movement and to estimate the traveled distance by analyzing the pattern of the movement [30].

As can be seen in Figure 2 (top), there are high frequency noise and spikes in the raw readings from the accelerometer. To smooth the raw measurements, 10th-order Butterworth low pass filter with a cut-off frequency is applied to them. The cut-off frequency is set to 3 Hz based on our experimental results obtained from 50 subjects between the ages of 25 and 35 with various walking speeds, and it can provide the step detection with high accuracy. The smoothed data after the filtering are represented in Figure 2 (bottom).

Afterwards, to identify the user step, the peak detection algorithm is applied to the smoothed acceleration data and then the peak values are found. The peaks represent the user steps. The result of the peak detection is illustrated by Figure 2 (bottom). The peak values are plotted as the red circles. Please refer to [18] for details of the peak detection algorithm.

**Figure 3:**
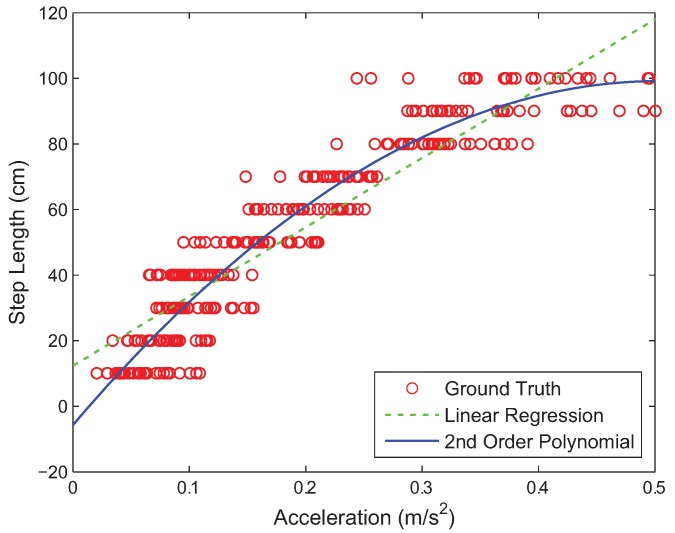
Relationship between the step length and acceleration magnitude obtained from the ground truth step data. The relationship is model by the polynomial regression or linear regression.

By analyzing the peak magnitudes of over 20,000 acceleration values that 50 users record every 10 cm, we could derive the relationship between the actual step length and the maximum peak value of the three-axis acceleration measurements, as shown in Figure 3. All the ground truth data points of the user steps in this figure are compared with the equation

(1) $$l=c_{1}p^{2}+c_{2}p+c_{3}$$

which is obtained by fitting them to the second order polynomial using polynomial regression, where *l* is the estimated step length, *p* is the maximum peak magnitude of the three-axis accelerations, and $c_{1}$, $c_{2}$, and $c_{3}$ are the polynomial coefficients. Our experiments show that $c_{1}=-412.93$, $c_{2}=416.24$, and $c_{3}=-5.71$ result in a high correlation between the actual step length and its estimated value *l* by the smartphone in the test environments described in this paper.

#### 4.1.2. Heading Determination

Heading information is available for improving the positioning accuracy in indoor environments. The heading data obtained from the accelerometer, gyroscope, and magnetometer can be easily affected by environment dynamics. Particularly, the magnetometer due to the magnetic interference may result in a noisy estimate of the user heading indoors. Therefore, we use the heading value provided by the CMDeviceMotion class of the iOS developer library [31] to obtain a reliable value of the user’s orientation. This value obtained by the accelerometer and gyroscope provides the most available and calibrated orientation information for the development of the iPhone application. Our method can also be conducted on an Android system using the Android developer library [32] that can offer a reliable value of user’s heading. We aim to estimate the location of the user using the sensory data (acceleration, heading angle, and RSS data) with uncertainty from the smartphone and the SKPF algorithm, but not to develop a novel approach to the heading inference.

### 4.2. Estimation of the Positional Measurement

**Figure 4:**
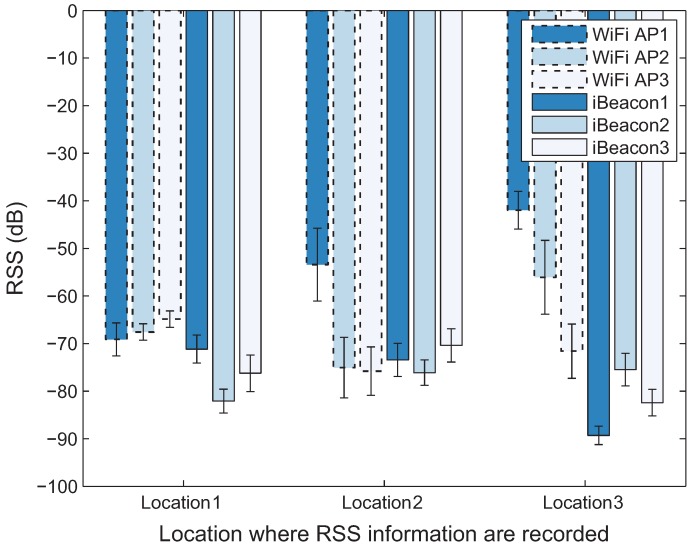
RSS comparison among adjacent locations.

The GPS operates poorly due to weak and blocked satellite signals in indoor environments. Therefore, it is difficult to obtain the measurement for the user’s location indoors. To determine the user location in such environments, there are many approaches based on alternative signals, such as acoustic, infrared, and RF. Since RF signals can easily be measured at receivers, such as WiFi and Bluetooth devices, many of the existing localization approaches leverage RSS fingerprints. Figure 4 represents the RSS values measured at adjacent locations (three orange circle points) in the test site described in Section 5. As can be seen in Figure 4, although the fluctuation in the RSS value exists, the value of the fingerprint can be used to distinguish the different locations, since the fingerprint of the same RF transmitter can change remarkably at each location in the area of interest. Because of this property, the fingerprints can also be used to estimate the position of the user located within the area of interest.

In our localization system, the machine learning algorithm is employed to determine the location of the user using the RSS fingerprints. We do not design a new machine learning algorithm but leverage only the conventional algorithms to provide the information about the user location, such as ANN (artificial neural network), KNN (*k*-nearest neighbors algorithm), NBC (naive Bayes classifier), and SVM (support vector machine). Hence, the machine learning algorithms used in this study are implemented with the machine learning library of the Open Source Computer Vision (OpenCV) written in optimized C/C++ [33].

The process to locate the user using the machine learning approach is divided into two phases: offline and online. In the offline phase, the RSS fingerprints from the WiFi APs and iBeacons are collected using the smartphone at selected locations in the area of interest (e.g., green squares and orange circles marked in Figure 7) and are sent to the server. Then, the machine learning algorithm on the server converts the recorded fingerprints (training data) into vectors (fingerprint database), i.e., the machine learning method builds the fingerprint map. During the site survey, we observed that RSS information is greatly affected by the heading direction of the user. Since the user’s body can be considered to be an obstruction similar to the wall between the RF transmitter and receiver, it may result in the fluctuation in the radio signal. Therefore, in addition to the corresponding RSS fingerprint for each location, the user’s direction is also recorded. The heading information is obtained from the built-in sensors on the smartphone, as described in Section 4.1.2. The influence of the user’s direction will be discussed in Section 6.1. The resultant recorded information at the *i*th location can be expressed as a tuple of form $\left(RSSw_{m}^{i},RSSb_{n}^{i},h^{i}\right)$, where $RSSw_{m}$ is the RSS value of the *m*th WiFi AP, $RSSb_{n}$ is the RSS value of the *n*th iBeacon, and *h* is the user heading. During the online phase, when the smartphone client sends a location query with the information about the currently observed RSS and direction to the server, the machine learning approach on the server estimates the user’s location that best matches the observed information via fingerprint database and sends back the estimated position of the user to the smartphone. The estimated value is used as a positional measurement of the user in the SKPF algorithm. Thus, the positional measurement is estimated using the machine learning instead of GPS.

### 4.3. Pedestrian Model

**Figure 5:**
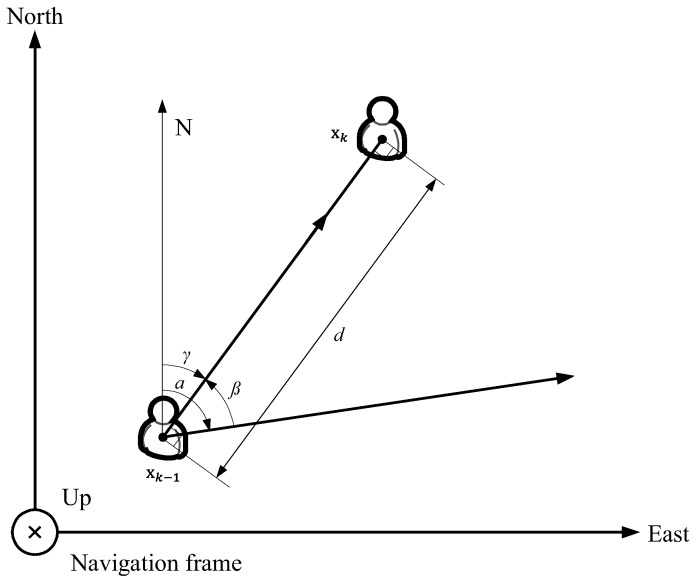
Pedestrian model. This model just considers *x*-axis and *y*-axis position of the positioning system user in the navigation frame.

The pedestrian model used to account for the motion of user in our positioning system is shown in Figure 5. The state of the pedestrian at time *k* can be represented as the vector $x_{k}={\left[\begin{array}{cc} x_{k} & y_{k} \end{array}\right]}^{T}$, where $\left(x_{k},y_{k}\right)$ is *x*-axis and *y*-axis position in the navigation frame. The navigation frame is the local geodetic frame where the *x*-axis, *y*-axis, and *z*-axis indicate east, true north, and up direction, respectively. Note that *z*-axis point is not considered for land-pedestrian navigation. Therefore, the state $x_{k}$ has only the *x*-axis and *y*-axis positional information. In this model, the angles α and γ indicate the heading angle of the user at times k−1 and *k*, respectively, and the angle β is defined as the counterclockwise angle between state vectors $x_{k-1}$ and $x_{k}$. The value of *d* represents the traveled distance of the pedestrian between time steps k−1 and *k*.

In the pedestrian model used to represent the pedestrian motion with INS sensors, such as accelerometer and gyroscope, the position of the pedestrian can be denoted by

(2) $$\begin{array}{ccc} x_{k} & = & x_{k-1}+d\sin\left(\alpha \right)\cos\left(\beta \right)-d\cos\left(\alpha \right)\sin\left(\beta \right)\; \end{array}$$

(3) $$\begin{array}{ccc} y_{k} & = & y_{k-1}+d\sin\left(\alpha \right)\sin\left(\beta \right)+d\cos\left(\alpha \right)\cos\left(\beta \right)\; \end{array}$$

where *d* is calculated by the maximum peak value of the three-axis accelerations between time steps k−1 and *k*, and both α and β are determined by accelerometer and gyroscope, as described in Section 4.1.

Applying the sine and cosine rule to Equations (2) and (3) and substituting α−β with γ, both Equations (2) and (3) can be expressed as

(4) $$\begin{array}{ccc} x_{k} & = & x_{k-1}+d\sin\left(\gamma \right) \end{array}$$

(5) $$\begin{array}{ccc} y_{k} & = & y_{k-1}+d\cos\left(\gamma \right). \end{array}$$

By assuming that process noise $w_{k-1}$ and measurement noise $v_{k}$ are all zero-mean white noise with covariance $Q_{k-1}$ and $R_{k}$, respectively, the system and measurement model of the pedestrian motion can be determined by

(6) $$\begin{array}{ccc} x_{k} & = & F_{k-1}x_{k-1}+G_{k-1}d+w_{k-1} \end{array}$$

(7) $$\begin{array}{ccc} z_{k} & = & H_{k}x_{k}+v_{k} \end{array}$$

where $G_{k-1}={\left[\begin{array}{cc} \sin(\gamma ) & \cos(\gamma ) \end{array}\right]}^{T}$, and $F_{k-1}$ is 2×2 identity matrix. In Equation (7), the value of $z_{k}$ represents the positional measurement ${\left[\begin{array}{cc} x_{k} & y_{k} \end{array}\right]}^{T}$ obtained by the machine learning and RSS information from the RF receivers, as described in Section 4.2, where $\left(x_{k},y_{k}\right)$ is *x*-axis and *y*-axis position in the navigation frame. Since the measurement $z_{k}$ is the same space as the model state $x_{k}$, $H_{k}$ is 2×2 identity matrix. Moreover, the measurement noise $v_{k}$ denotes position estimation error generated by the machine learning algorithm.

### 4.4. Sigma-Point Kalman Particle Filter (SKPF)

#### 4.4.1. Basic Idea

In the positioning system, the Bayesian filters have been used to integrate sensory data from all different sources to obtain better positioning accuracy. Among Bayesian filters, the Kalman filter (KF) and its variants, including the unscented Kalman filter (UKF), have been widely used for the navigation systems, since they are efficient in terms of the computational cost while providing a high accuracy in localization [9,28]. Recently, various indoor positioning approaches using PF have been proposed to achieve higher positioning accuracy than KF and its variants in indoor environments [12,29]. However, since PF requires many particles (samples) for the high positioning performance, it can lead to considerable computational cost compared to KF and its variants.

In this paper, we propose the enhanced KF called a sigma-point Kalman particle filter (SKPF), which can achieve better positioning performance compared with KF, UKF, and PF by leveraging both the unscented transformation (UT) of UKF and the weighting method of PF. The SKPF algorithm can provide a balance between the low computational effort of UKF and the high estimation performance of PF when estimating the state (the location of the user) of the positioning and tracking system as follows:

- The SKPF algorithm exploits the unscented transformation (UT) used in UKF. This enables the SKPF approach to estimate the state of the system using a small number of samples, while the PF relies on many samples to achieve accurate results. Hence, the SKPF can provide higher computational efficiency compared with PF. This is addressed further in Section 4.4.1.1, Section 4.4.1.2 and Section 6.3.
- Unlike UKF that employs samples with the uniform weight for all system dynamics, the SKPF algorithm uses samples that have the different weight through the weighting method of PF, which evaluates the samples’ weights using the likelihood function proportional to the Gaussian/non-Gaussian posterior density. Consequently, the SKPF approach can offer higher estimation accuracy than KF and UKF and competitive estimation performance compared to PF. This is addressed in more detail in Section 4.4.1.3 and Section 6.2.

The SKPF algorithm can be summarized as follows. First, to describe the canonical form of the SKPF algorithm that can be applied to the nonlinear systems without the linearization for the nonlinear functions using the unscented transform (UT), suppose we have a nonlinear discrete-time system with *n*-dimensional state vector $x_{k}$ given as follows:

(8) $$\begin{array}{ccc} x_{k} & = & f\left(x_{k-1},u_{k-1}\right)+w_{k-1} \end{array}$$

(9) $$\begin{array}{ccc} z_{k} & = & h\left(x_{k}\right)+v_{k} \end{array}$$

where $x_{k}$ and $u_{k}$ indicate the *n*-dimensional state vector and the control vector at time *k*, respectively. The process equation f(·) means the state transition model which is applied to the previous state $x_{k-1}$, and the measurement equation h(·) denotes the observation operator that maps the true state space into the observed space. They define the nonlinear system. The process noise $w_{k}$ and measurement noise $v_{k}$ are white, zero-mean, and uncorrelated, and have covariance matrices $Q_{k}$ and $R_{k}$, respectively. Our goal is to estimate the state $x_{k}$ based on our knowledge of the system dynamics and the availability of the noisy measurement $z_{k}$.

In the SKPF algorithm, the estimates of the state $x_{k}$ are represented by the following a posteriori and a priori values.

The a posteriori state estimate ${\hat{x}}_{k|k}$ denotes the expected value of $x_{k}$ conditioned on all of the measurements up to and including time *k*:

(10) $${\hat{x}}_{k|k}=E\left[x_{k}|z_{1},z_{2},\ldots ,z_{k}\right].$$

On the contrary, the a priori state estimate ${\hat{x}}_{k|k-1}$ denotes the expected value of $x_{k}$ conditioned on all of the measurements before time *k*:

(11) $${\hat{x}}_{k|k-1}=E\left[x_{k}|z_{1},z_{2},\ldots ,z_{k-1}\right].$$

We employ the term $P_{k}$ to indicate the covariance of the state estimate. $P_{k|k-1}$ and $P_{k|k}$ indicate the covariance of ${\hat{x}}_{k|k-1}$ and ${\hat{x}}_{k|k}$, respectively.

The state estimate and its covariance of SKPF is initialized before any measurements are available. Thus,

(12) $$\begin{array}{ccc} {\hat{x}}_{0|0} & = & E[x_{0}] \end{array}$$

(13) $$\begin{array}{ccc} P_{0|0} & = & E[\left(x_{0}-{\hat{x}}_{0|0}\right){\left(x_{0}-{\hat{x}}_{0|0}\right)}^{T}]. \end{array}$$

A set of 2n weighted sigma points (samples) used to estimate the state and covariance in SKPF can be written as ${\left\{x_{k}^{i},w_{k}^{i}\right\}}_{i=1}^{2n}$, where $\left\{x_{k}^{i},i=1,\ldots ,2n\right\}$ is a set of samples, and $\left\{w_{k}^{i},i=1,\ldots ,2n\right\}$ represents weights for each of the samples. The sum of weights is 1 such that $\sum_{i}w_{k}^{i}=1$.

**Figure 6:**
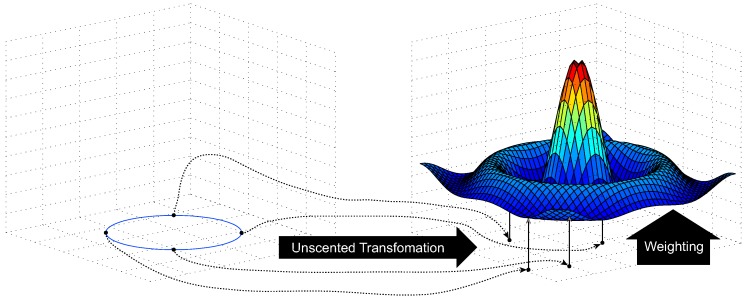
The principle of the SKPF algorithm.

The principle of the SKPF algorithm is shown in Figure 6. The unscented transformation (UT) is designed on the intuition that it is easier to approximate a probability distribution than it is to approximate an arbitrary nonlinear function using the linearization [34,35]. To overcome the deficiencies of linearization for the nonlinear function, the UT propagates the state estimate and its covariance through nonlinear transformations. The SKPF algorithm works in a two-phase process: a prediction (Section 4.4.1.1) and an update (Section 4.4.1.2).

##### *(1) Prediction* 

In the prediction phase, the state $x_{k}$ of the system at time *k* is predicted through the UT for sigma points (samples) drawn from the a posteriori estimated state ${\hat{x}}_{k-1|k-1}$ at time k−1. The predicted state at time *k* means the a priori estimated state ${\hat{x}}_{k|k-1}$. The following equations are employed to propagate the state estimate ${\hat{x}}_{k-1|k-1}$ and its covariance $P_{k-1|k-1}$ from time k−1 to *k* using the UT. First, 2n samples $x_{k-1|k-1}^{i}$ with associated weights $w_{k-1}^{i}$ around the estimate ${\hat{x}}_{k-1|k-1}$ are selected:

$$x_{k-1|k-1}^{i}={\hat{x}}_{k-1|k-1}+{\left(\sqrt{nP_{k-1|k-1}}\right)}_{i},\;i=1,\ldots ,n$$

(14) $$x_{k-1|k-1}^{i}={\hat{x}}_{k-1|k-1}-{\left(\sqrt{nP_{k-1|k-1}}\right)}_{i-n},\;i=n+1,\ldots ,2n$$

(15) $$w_{k-1}^{i}=w_{k}^{i}=\frac{1}{2n},\;i=1,\ldots ,2n$$

where $\sqrt{nP_{k-1|k-1}}$ is the matrix square root of $nP_{k-1|k-1}$ such that $\left(\sqrt{nP_{k-1|k-1}}\right){\left(\sqrt{nP_{k-1|k-1}}\right)}^{T}=nP_{k-1|k-1}$, and ${\left(\sqrt{nP_{k-1|k-1}}\right)}_{i}$ is the *i*th column (or row) of $\sqrt{nP_{k-1|k-1}}$. The Cholesky factorization can be used to calculate the matrix square root.

The nonlinear system equation f(·) is used to transform the samples $x_{k-1|k-1}^{i}$ into $x_{k|k-1}^{i}$ vectors as follows:

(16) $$x_{k|k-1}^{i}=f\left(x_{k-1|k-1}^{i},u_{k-1}^{i}\right),\;i=1,\ldots ,2n.$$

The a priori state estimate ${\hat{x}}_{k|k-1}$ at time *k* is determined by the weighted sum of the transformed samples:

(17) $${\hat{x}}_{k|k-1}=\sum_{i=1}^{2n}w_{k}^{i}x_{k|k-1}^{i}.$$

The covariance of the estimation error of ${\hat{x}}_{k|k-1}$ is the weighted outer product of the transformed samples. Note that the covariance $Q_{k-1}$ is added to the end of the equation to take the process noise into consideration:

(18) $$P_{k|k-1}=\sum_{i=1}^{2n}w_{k}^{i}\left(x_{k|k-1}^{i}-{\hat{x}}_{k|k-1}\right){\left(x_{k|k-1}^{i}-{\hat{x}}_{k|k-1}\right)}^{T}+Q_{k-1}.$$

##### *(2) Update* 

In the update phase, when the measurement $z_{k}$ of the state $x_{k}$ at time *k* is observed, the predicted state can be corrected using the measurement. The following equations are used to update the predicted state estimate ${\hat{x}}_{k|k-1}$ and its covariance $P_{k|k-1}$ at time *k* using the UT and measurement $z_{k}$. First, a set of samples $x_{k|k-1}^{i}$ is derived from ${\hat{x}}_{k|k-1}$ and $P_{k|k-1}$, since the a priori statistics are the current best guess for the mean and covariance of $x_{k}$. Thus,

(19) $$\begin{array}{ccc} x_{k|k-1}^{i} & = & {\hat{x}}_{k|k-1}+{\left(\sqrt{nP_{k|k-1}}\right)}_{i},\;i=1,\ldots ,n\\ x_{k|k-1}^{i} & = & {\hat{x}}_{k|k-1}-{\left(\sqrt{nP_{k|k-1}}\right)}_{i-n},\;i=n+1,\ldots ,2n. \end{array}$$

The nonlinear measurement equation h(·) is employed to transform the samples $x_{k|k-1}^{i}$ into $z_{k}^{i}$ vectors (predicted measurements) as follows:

(20) $$z_{k}^{i}=h\left(x_{k|k-1}^{i}\right),\;i=1,\ldots ,2n.$$

The estimate of the measurement ${\hat{z}}_{k}$ at time *k* can be determined by the weighted sum of the transformed samples. Therefore,

(21) $${\hat{z}}_{k}=\sum_{i=1}^{2n}w_{k}^{i}z_{k}^{i}.$$

The covariance of the measurement residual (innovation) $S_{k}$ is obtained from the weighted outer product of the transformed samples. Note that the covariance $R_{k}$ is added at the end of the equation to take account of the measurement noise. Thus,

(22) $$S_{k}=\sum_{i=1}^{2n}w_{k}^{i}\left(z_{k}^{i}-{\hat{z}}_{k}\right){\left(z_{k}^{i}-{\hat{z}}_{k}\right)}^{T}+R_{k}.$$

Then, the cross covariance between ${\hat{x}}_{k|k-1}$ and ${\hat{z}}_{k}$ is given by

(23) $$P_{\mathrm{xz}}=\sum_{i=1}^{2n}w_{k}^{i}\left(x_{k|k-1}^{i}-{\hat{x}}_{k|k-1}\right){\left(z_{k}^{i}-{\hat{z}}_{k}\right)}^{T}.$$

The optimal Kalman gain $K_{k}$ for the state estimate is determined by the normal KF equations as follows:

(24) $$K_{k}=P_{\mathrm{xz}}S_{k}^{-1}.$$

The a posteriori state estimate ${\hat{x}}_{k|k}$ at time *k* is obtained from the reweighted set of the samples given by Equation (19) through the weighting approach of PF. This step is addressed in more details in the next section.

Assuming that the a posteriori state estimate ${\hat{x}}_{k|k}={\hat{x}}_{k|k-1}+K_{k}\left(z_{k}-{\hat{z}}_{k}\right)$ computed by the KF approximates ${\hat{x}}_{k|k}$ obtained by PF, the a posteriori error covariance $P_{k|k}$ is also calculated using the normal KF equation. Thus,

(25) $$P_{k|k}=P_{k|k-1}-K_{k}S_{k}K_{k}^{T}.$$

##### *(3)* A posteriori *State Estimate*

In the SKPF approach, the estimate of the a posteriori state is obtained using PF. The PF is a sequential Monte Carlo method that recursively estimate the sequence of system states from measurement. This approximates the posterior density $p(x_{k}|z_{1:k})$ of Bayesian filter using a set of 2n weighted samples (i.e., particles) determined by

(26) $${\left\{x_{k}^{i},w_{k}^{i}\right\}}_{i}^{2n}∼p\left(x_{k}|z_{1:k}\right)$$

where $\left\{x_{k}^{i},i=1,\ldots ,2n\right\}$ is a set of samples at time *k*, $\left\{w_{k}^{i},i=1,\ldots ,2n\right\}$ is the importance weights for each of the samples. The sum of weights is 1 such that $\sum_{i}w_{k}^{i}=1$. Then, the posterior pdf approximated with weights is given by

(27) $$p\left(x_{k}|z_{1:k}\right)\approx \sum_{i=1}^{2n}w_{k}^{i}\delta \left(x_{k}-x_{k}^{i}\right)$$

where δ(·) indicates the Dirac delta measure.

The weights for each sample are given by the principle of importance [36]. Thus

(28) $$w_{k}^{i}\propto w_{k-1}^{i}\frac{p\left(z_{k}|x_{k}^{i}\right)p\left(x_{k}^{i}|x_{k-1}^{i}\right)}{q(x_{k}^{i}|x_{0:k-1}^{i},z_{1:k})}$$

where $p(z_{k}|x_{k}^{i})$ is the relative likelihood of each particle $x_{k}^{i}$ conditioned on the measurement $z_{k}$, $q(x_{k}^{i}|x_{0:k-1}^{i},z_{1:k})$ is the proposal distribution (importance density), and $p(x_{k}^{i}|x_{k-1}^{i})$ denotes the prior density.

Since it is intuitive and simple to implement, the most PF algorithms choose the importance distribution to be the prior density as follows [37]:

(29) $$q\left(x_{k}^{i}|x_{0:k-1}^{i},z_{1:k}\right)=p\left(x_{k}^{i}|x_{k-1}^{i}\right).$$

Substituting Equation (29) into Equation (28) gives

(30) $$w_{k}^{i}\propto w_{k-1}^{i}p\left(z_{k}|x_{k}^{i}\right).$$

In the SKPF algorithm, however, noting that the unscented transformation is applied at every time step, we have $w_{k-1}^{i}=1/2n$∀i as given by Equation (15) in the prediction phase. Therefore, the weights of samples by the weighting method of PF can be recalculated as

(31) $$w_{k}^{i}\propto p\left(z_{k}|x_{k|k-1}^{i}\right)$$

where $x_{k|k-1}^{i}$ is the sample obtained from (19).

After the measurement $z_{k}$ is observed at time *k*, the conditional relative likelihood $p(z_{k}|x_{k|k-1}^{i})$ of each sample $x_{k|k-1}^{i}$ can be calculated. This can be performed if we know the nonlinear measurement equation and the pdf of the measurement noise. For example, if the *m*-dimensional measurement equation is given as Equation (9) and the measurement noise $v_{k}∼N\left(0,R_{k}\right)$, then the relative likelihood of each particle $x_{k|k-1}^{i}$ can be determined by

(32) $$p\left(z_{k}|x_{k|k-1}^{i}\right)=P\left[v_{k}=z_{k}-h\left(x_{k|k-1}^{i}\right)\right].$$

Using Equation (20) and the pdf of the measurement noise $v_{k}$, the relative likelihood can be expressed as

(33) p(zk|xk|k−1i)=P[vk=zk−zki]p(zk|xk|k−1i)∝1(2π)m/2|Rk|1/2exp−zk−zkiTRk−1zk−zki2.

The resultant weight of each sample can be determined by Equation (33), and it is normalized to ensure that the sum of all the weights is equal to one as follows:

(34) $$w_{k}^{i}=\frac{w_{k}^{i}}{\sum_{i=1}^{2n}w_{k}^{i}}.$$

Using the samples $x_{k|k-1}^{i}$ in Equation (19) and their normalized weights $w_{k}^{i}$ obtained by Equation (34), the a posteriori state estimate is given by the weighted sum. Thus,

(35) $${\hat{x}}_{k|k}=\sum_{i=1}^{2n}w_{k}^{i}x_{k|k-1}^{i}.$$

#### 4.4.2. SKPF-Based Localization Algorithm

A pseudo-code description of the pedestrian positioning approach based on the SKPF algorithm is given by Algorithm 1. The user movement for indoor environments in the position method can be described by two-dimensional state space that consists of positional information $x_{k}={\left[\begin{array}{cc} x_{k} & y_{k} \end{array}\right]}^{T}$ at time *k* through the pedestrian model introduced in Section 4.3. Therefore, during the prediction (dead reckoning) step of this algorithm, the state of the user is predicted using both the samples transformed by the system matrix *F* and the step length and heading of the user obtained from inertial sensors (Section 4.1). The update phase corrects the predicted state of the user through both the samples propagated by the measurement matrix *H* and the positional measurement $z_{k}$ obtained from the fingerprinting method (Section 4.2).

**Algorithm 1** SKPF-based Localization Algorithm
$\left[{\left\{x_{k}^{i},w_{k}^{i}\right\}}_{i=1}^{2n}\right]=\mathrm{SKPF}\left[{\left\{x_{k-1}^{i},w_{k-1}^{i}\right\}}_{i=1}^{2n},z_{k}\right]$

Initialize SKPF ${\hat{x}}_{0|0}=E\left[x_{0}\right]$ $P_{0|0}=E\left[\left(x_{0}-{\hat{x}}_{0|0}\right){\left(x_{0}-{\hat{x}}_{0|0}\right)}^{T}\right]$Estimate the state and error covariance at every time step−Prediction (dead reckoning)·Determine $x_{k-1|k-1}^{i}$ and $w_{k-1}^{i}$ using (14) and (15)·Propagate the samples $x_{k-1|k-1}^{i}$ using (6) **for** i=1:2n **do** $x_{k|k-1}^{i}=F_{k-1}x_{k-1|k-1}^{i}+G_{k-1}d$ **end for**·Calculate ${\hat{x}}_{k|k-1}$ and $P_{k|k-1}$ from (17) and (18)−Update·Select the samples $x_{k|k-1}^{i}$ using (19)·Propagate $x_{k|k-1}^{i}$ using (7) **for** i=1:2n **do** $z_{k}^{i}=H_{k}x_{k|k-1}^{i}$ **end for**·Compute ${\hat{z}}_{k}$ and $S_{k}$ according to (21) and (22)·Determine $P_{\mathrm{xz}}$ between ${\hat{x}}_{k|k-1}$ and ${\hat{z}}_{k}$ from (23)·Calculate the optimal Kalman gain $K_{k}$ using (24)·Evaluate $w_{k}^{i}$ using $z_{k}$ according to (33)·Normalize weights $w_{k}^{i}$ using (34)·Calculate ${\hat{x}}_{k|k}$ and $P_{k|k}$ from (35) and (25)


## 5. Experimental Testbed

**Figure 7:**
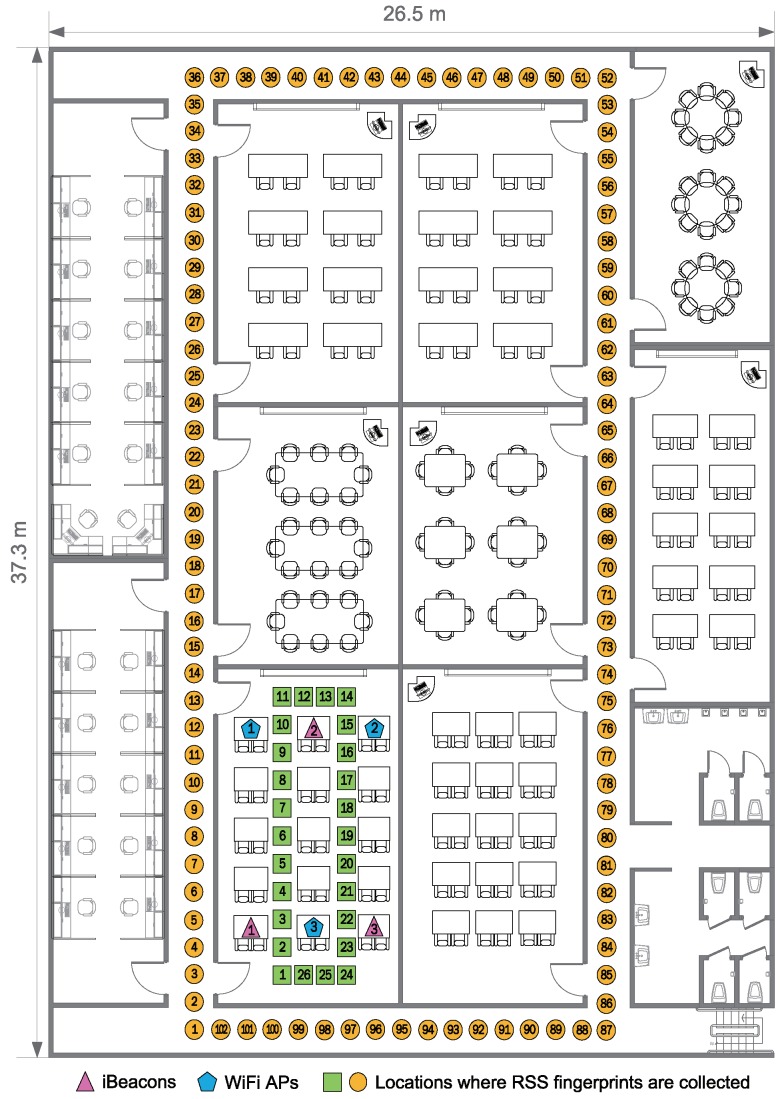
Floor plan of the test site where the experiments were carried out. RSS information is recorded in the hallways and inside the lecture room.

This section describes the testbed configuration that is used to build the RSS fingerprint database and to evaluate the performance of our positioning algorithm. Our experimental site was located on the first floor of the International Center for Converging Technology in the Korea University. The layout of the floor is shown in Figure 7. The area of the test site is about 37.3 m by 26.5 m.

In our experiments, the wireless network was comprised of three WiFi APs (ipTime N104T) and three iBeacons (Estimote) that work in 2.4 GHz ISM band. The position of the RF transmitters deployed in the lecture room is represented by pink triangles and blue pentagons marked with a sequence number in Figure 7. The iBeacons operate via the Bluetooth Low Energy (BLE) technology, which requires a low transmit power of 10 mW and has a maximum bit rate of 1 Mbps and a transmission range of 100 m. The mobile host used to collect RSS information from both WiFi APs and iBeacons and to estimate the user’s position was a smartphone (iPhone 5S) equipped with inertial sensors, such as a three-axis gyroscope and accelerometer, and wireless adapters for WiFi 802.11n and Bluetooth 4.0. The update rate of the accelerometer and gyroscope on the smartphone is 100 Hz, and the update interval of WiFi and iBeacon receiver on the phone is 1 s.

50 users between the ages of 25 and 35 with a variety of walking speeds took part in our experiments. Both green square and orange circle symbols in Figure 7 represent the physical locations where the location sample that consists of RSS fingerprints from both WiFi APs and iBeacons as well as heading information are collected by the user with the mobile phone during the offline phase of the fingerprinting method described in Section 4.2. They are deployed at intervals of one meter with the label (sequence number) of the physical location in the hallways and inside the lecture room. To construct the fingerprint map (database), we collected more than 100 location samples at each physical position.

Since the RSS data from the same RF transmitter can vary significantly at the different locations due to obstructions between the RF transmitter and receiver, to investigate the impact of the obstacles (due to the building structure) on RSS information at a given location, all the WiFi APs and iBeacons in our experiments were intentionally located in the same space. Based on these RF transmitters, our experiments can be classified into two testbeds depending on the test site: Scenarios S1 and S2.

In Scenario S1, the pedestrian with mobile device walks along the locations of green square symbols shown in Figure 7 in a clockwise direction inside the lecture room where WiFi APs and iBeacons are located. The testbed represents the wireless environment with good signal condition for WiFi APs and iBeacons, as there are no walls. In Scenario S2, the user walks along the hallways where orange circle symbols represented in Figure 7 are located in a clockwise direction. The testbed reflects the poor wireless environment in which the signals between the transmitter (WiFi APs and iBeacons) and the receiver (mobile host) are frequently or completely blocked due to many obstructions, such as walls.

**Figure 8:**
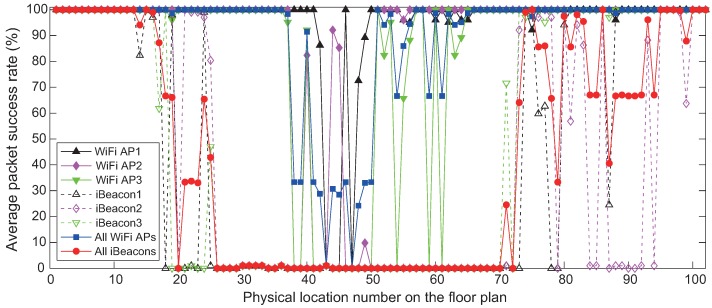
Average value of packet success rate measured by the mobile phone of 50 users at each physical location on the floor plan in the hallway Scenario S2.

Figure 8 represents the average value of packet success rate (PSR) obtained by RSS values received from all WiFi APs and iBeacons at each physical location in Scenario S2 with poor wireless environments (the orange circle represented in Figure 7). As can be seen in Figure 8, the average value of PSR for the remote location from WiFi AP and iBeacon is less than that for the location close to WiFi AP and iBeacon. The iBeacons in the experiments actually have a transmission range of about 25 m, since their RSS signals can be blocked by obstacles. In Scenario S2, the average value of PSR from all the WiFi APs and iBeacons for every position is about 89.76% and 44.32%, respectively. In contrast, for every physical location of Scenario S1 with good signal condition (the green square in Figure 7), our experimental results (not reported here) show that the average value of PSR from all the WiFi APs and iBeacons is 100% together.

## 6. Experimental Results

The following sections describe the experimental results for our localization approach in a real environment.

### 6.1. Evaluation of Positional Measurement Estimation

In this section, we analyze the performance of machine learning algorithms employed to build the fingerprint map (database) using the heading information of user and RSS values obtained from WiFi APs and iBeacons, and accordingly to estimate the positional measurement of the user based on the map. The machine learning algorithms include ANN, KNN, NBC, and SVM introduced in Section 4.2. In the ANN algorithm, the input layer has a node (or neuron) per input feature of the dataset, and the output layer has a node per class label [38]. Since the location sample used in our experiments has seven input features (three WiFi RSS values, three iBeacon RSS values, and one heading information), the input layer of the ANN algorithm in our experiments also has seven neurons. Since the number of the physical locations in Scenarios S1 and S2 is 26 and 102, respectively, the output layer of the ANN method also has 26 and 102 neurons in Scenarios S1 and S2, respectively. We estimated the best parameters for the SVM algorithm through iterative cross validations using the SVM training function *train_auto* of OpenCV [33]. We used the NBC algorithm with its default parameters provided by OpenCV and the KNN algorithm with k=3 nearest neighbors, which yield the best results of the KNN approach in Scenarios S1 and S2.

We assume that the fingerprint map has already been built through the offline phase before the online step of the fingerprinting approach. Given the *i*th physical location $z_{p}^{i}$ and estimated physical location (positional measurement) $z^{i}$ from the fingerprint map when the location samples (mentioned in Section 5) that consist of RSS values and heading information are measured at the physical location $z_{p}^{i}$ during the online phase, we define the location mapping as a function

(36) $$m(z^{i})=\left\{\begin{array}{ccc} 1 & z^{i}=z_{p}^{i} & i=1,\ldots ,N_{z_{p}}\\ 0 & otherwise \end{array}\right.$$

where $z_{p}^{i}$ and $z^{i}$ are one of the physical locations (green square symbols used in Scenario S1 or orange circle symbols used in Scenario S2) shown in Figure 7, and $N_{z_{p}}$ is the number of the physical locations in Scenario S1 or S2. Using Equation (36), the location mapping rate MR can be expressed as a percentage of the number of the estimated physical location $z^{i}$ that match the *i*th physical location $z_{p}^{i}$ for $N_{z_{p}}$ physical locations. Therefore

(37) $$\mathrm{MR}=\frac{1}{N_{z_{p}}}\sum_{i=1}^{N_{z_{p}}}m\left(z^{i}\right)\times 100\%.$$

We used the average value of mapping rate determined by the mobile phone of 50 users in our experiments.

**Figure 9:**
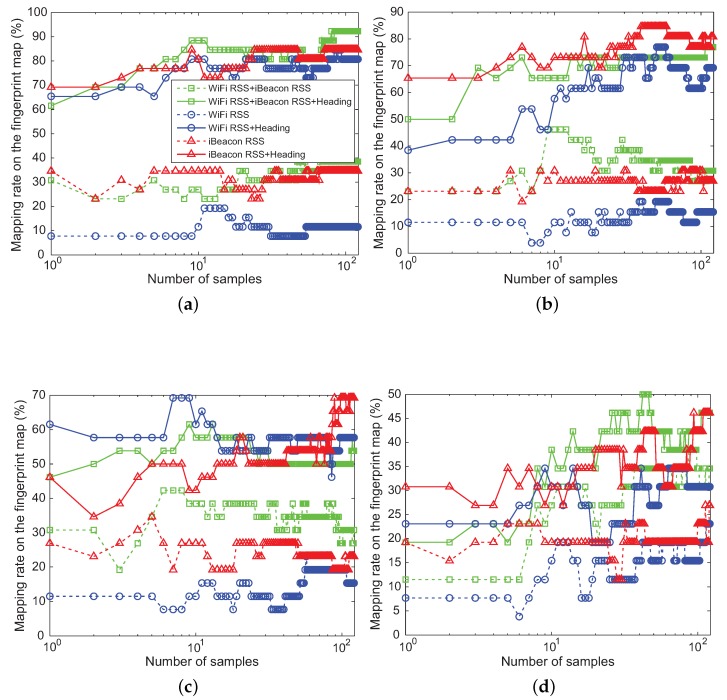
Mapping rate of: (**a**) NBC; (**b**) KNN; (**c**) ANN; and (**d**) SVM in Scenario S1 with good wireless signal condition.

**Figure 10:**
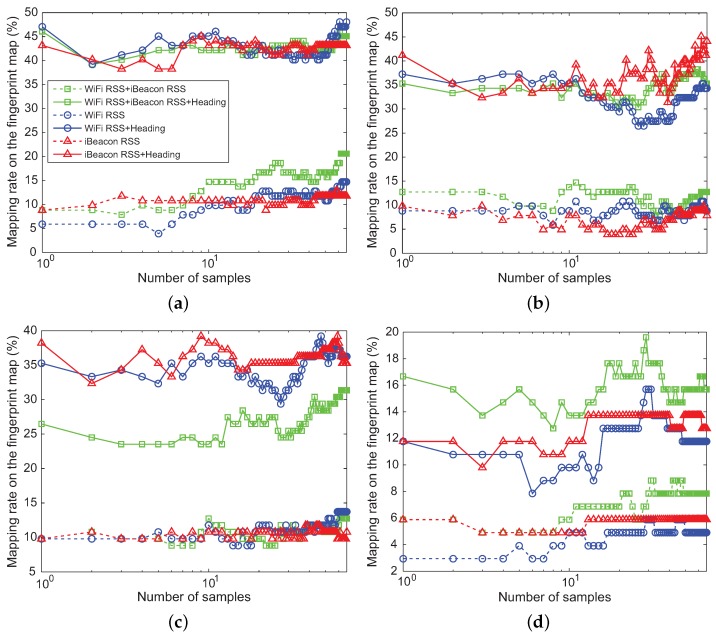
Mapping rate of: (**a**) NBC; (**b**) KNN; (**c**) ANN; and (**d**) SVM in Scenario S2 with poor wireless signal condition.

Figure 9 and Figure 10 show the average value of mapping rate calculated in Scenarios S1 and S2, respectively. Scenario S1 with good signal condition (high PSR) has a higher mapping rate than Scenario S2 with poor wireless environments (low PSR). The mapping rate are not greatly affected by kinds of the RSS features (i.e., iBeacon RSS, WiFi RSS, and both iBeacon RSS and WiFi RSS) in both scenarios. However, when the heading of the user is applied for the machine learning algorithm, the mapping rate is significantly improved compared with when not using the heading information.

In Scenario S1, as the number of location samples measured in the online phase increases, the mapping rate can reach a higher value. However, since much time is spent collecting the samples, the real-time process of the position estimate is not feasible. On the contrary, in Scenario S2, the ratio of the mapping is not affected by the number of the samples during the online step. Therefore, only one location sample was used for the real-time positioning of the user in our experiments.

**Figure 11:**
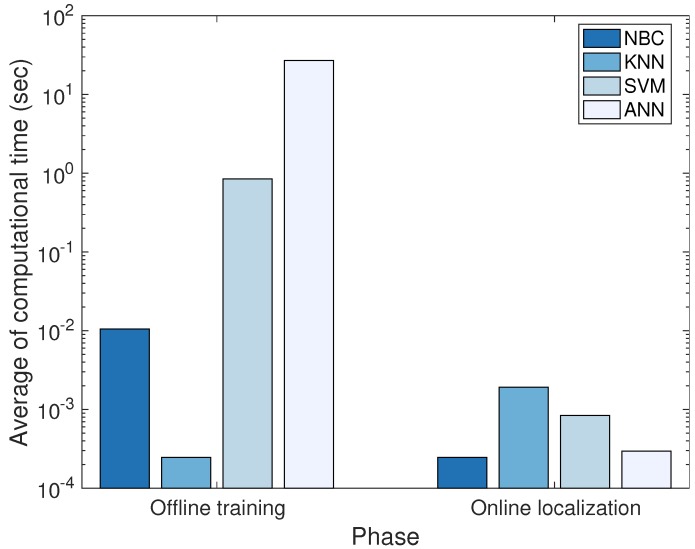
Average execution time of offline and online phase in machine learning algorithms.

Figure 11 represents the average time in seconds required to construct the fingerprint map with 1536 location samples in the offline phase and the one spent to estimate the positional measurement of the user in the online phase for each of the machine learning algorithms. As can be noticed, even though NBC requires a little more execution time than KNN in the offline phase, it spends less execution time than different machine learning algorithms during the offline and online phases. Figure 9 and Figure 10 also indicate that NBC achieves better performance than other machine learning algorithms in terms of the mapping rate. Hence, the position estimated by NBC instead of GPS is used as a measurement $z_{k}$ for SKPF at time *k* in our positioning system.

### 6.2. Positioning Accuracy

The SKPF algorithm proposed in this paper is evaluated through empirical tests to verify the validity of it as an indoor position estimator. During our experiments carried out for the evaluation, the users moved along physical locations marked with a sequence number in Scenarios S1 and S2, and then their position was estimated by the SKPF algorithm.

Table 1 summarizes the main features and notations for the positioning methods used in our experiments. In the method P as a dead reckoning (DR), the position of the user is predicted using the sensory data (acceleration and heading) of the smartphone. Instead of GPS, the positional measurement of the user can be obtained from the NBC-based fingerprinting method mentioned in Section 6.1. However, there are errors in positional information obtained from both the DR and fingerprinting approach. The SKPF algorithm is used to update the position of the user by integrating the positional data obtained from both fingerprinting and DR with uncertainty. According to the kinds of training data used in the NBC-based fingerprinting method, the SKPF algorithm is classified into three operational modes: PU1 (iBeacon RSS and heading), PU2 (WiFi RSS and heading), and PU3 (iBeacon RSS, WiFi RSS, and heading). To analyze the positioning performance of the SKPF algorithm in methods P, PU1, PU2, and PU3, the SKPF algorithm is replaced with the conventional Bayes filters, such as KF, UKF, and PF.

**Figure 12:**
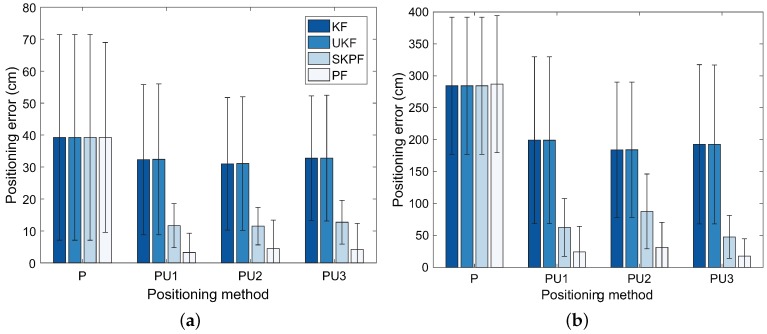
Performance of the localization algorithms for Scenarios (**a**) S1 and (**b**) S2 in terms of average and standard deviation of the positioning error.

Figure 12 indicates the mean and standard deviation of the localization error for each positioning algorithm executed by 50 users in Scenarios S1 and S2, respectively. As can be seen in these figures, the accuracy and reliability of the position estimate can be improved when both the prediction (dead reckoning) and update phase are used together in the Bayesian filtering compared with when only the prediction phase is exploited. Especially, in Scenario S2 with the poor wireless signal condition, the positioning accuracy is increased significantly. The localization results of PU1, PU2, and PU3 show that the positioning accuracy is not greatly affected by kinds of the RSS features for the measurement estimation.

The SKPF can provide higher accuracy of the position estimate than KF and UKF, as shown in Figure 12a,b. In Scenarios S1 and S2, the positioning algorithms (PU1, PU2, and PU3) based on SKPF has the average localization error of about 10.37 cm and 71.63 cm, respectively. In this case, the use of SKPF can achieve about 20.1% and 20.2% higher accuracy than KF and UKF in Scenario S1, respectively, and it can reach about 151% and 152% higher accuracy compared with KF and UKF in Scenario S2, respectively. Furthermore, as illustrated in Figure 12a,b, since the SKPF algorithm has the lower value of the positioning error standard deviation than other Bayes filters, it can execute a more reliable position estimate. This is because, unlike UKF that uses samples (sigma points) with the uniform weight for all system dynamics, SKPF employs samples that have the different weight through the weighting method of PF that evaluates the weight of the sample using the likelihood function proportional to the posterior density. Hence, the SKPF algorithm can offer better positioning performance than KF and UKF and competitive performance compared to PF.

**Figure 13:**
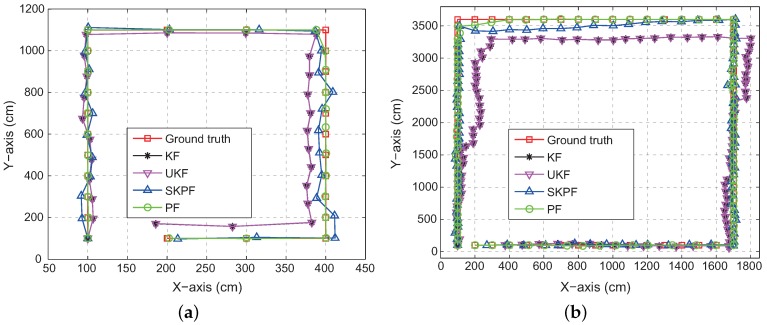
User trajectory estimated using localization algorithms when the user walks along physical locations marked with a sequence number in: (**a**) Scenario S1; and (**b**) Scenario S2.

We can analyze in more detail these errors by observing Figure 13, which represents the user trajectory estimated by the positioning algorithm where PU3 is applied for the Bayesian filter. In Scenario S1, the localization accuracy difference between SKPF and both KF and UKF is large. The improvements in the accuracy are much clearer in Scenario S2 with the poor signal environments, becoming particularly remarkable at the locations with the lowest PSR for all the WiFi APs and iBeacons (i.e., positions marked with 40 to 49 in Figure 8). According to these results, the positioning approach when SKPF is used is shown to be able to provide accurate and reliable positional information even in the complicated building structure and bad signal condition.

### 6.3. Computational Complexity and Time

For the computational complexity and memory requirement, KF and UKF scale in general O(m) and $O(n^{3})$, respectively, where *m* denotes the dimension of the measurement $z_{k}$ and *n* is the dimension of the state $x_{k}$ [39,40]. In our test environments, since the value of *m* is equal to the value of *n*, we can observe that KF has a less complex method and requires less memory than UKF. PF, which uses many particles (samples), requires substantial computational cost and memory usage to estimate the location of the user. Indeed, both these quantities scale as $O(N_{s})$, where $N_{s}$ denotes the number of the particles [41]. By contrast, since SKPF can estimate the position of the user with the same samples as UKF that uses a minimal set of samples, the computational complexity and memory requirement of SKPF scales as $O(n^{3})$.

**Figure 14:**
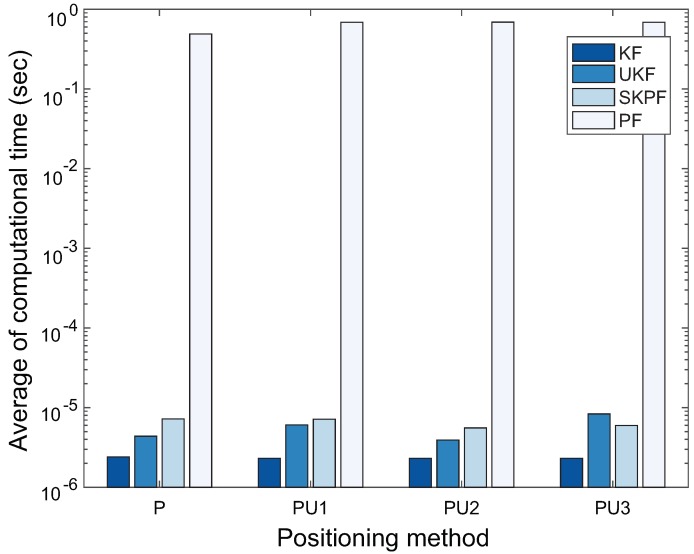
Average of computational time for each positioning method.

**Figure 15:**
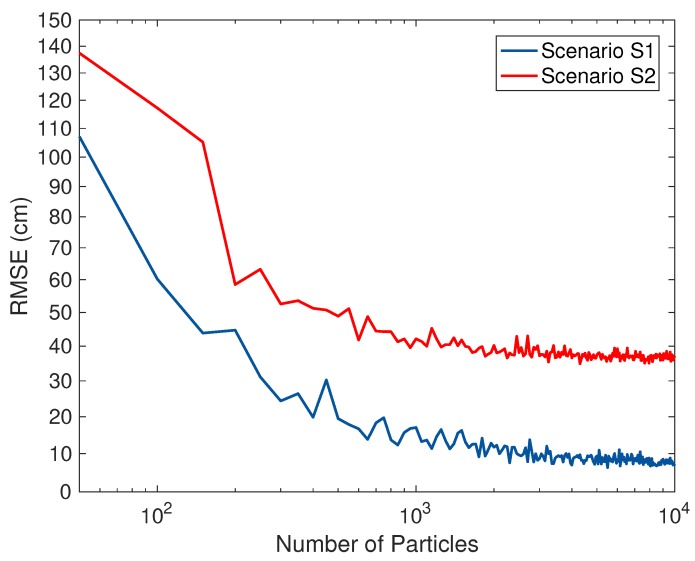
RMSE value between the estimated location by PF and its corresponding actual position versus the number of particles for PF in Scenarios S1 and S2.

Figure 14 represents the average computational time required for the positioning process in each of localization algorithms. As illustrated in this figure, even though the PF has the highest positioning accuracy among the Bayesian filters, the computational time of PF may not be appropriate for the real-time process of the localization. In our experiments, the PF employed $10^{3}$ particles for the positioning. For Scenarios S1 and S2, we decided the optimal number of particles for PF used in the positioning algorithms PU1, PU2, and PU3 by calculating the value of the root mean square error (RMSE) between the estimated location by PF and its corresponding actual position versus the number of particles, as shown in Figure 15. The RMSE value in the figure decrease abruptly until the number of particles reaches $10^{3}$ and then can converge to the value of about 10 cm for the Scenario S1 and about 40 cm for the Scenario S2, respectively. This means that the location of the pedestrian can be estimated most efficiently at the value of about $10^{3}$ particles, i.e., the optimal number of particles.

In contrast, although SKPF has slightly smaller positioning accuracy than PF, it can carry out faster localization. This is because the SKPF algorithm uses the unscented transformation (UT) of UKF. This enables the SKPF approach to estimate the position of the user with a small number of samples, while the PF depends on a large number of samples to achieve accurate results. Therefore, the SKPF algorithm can provide the higher computational efficiency compared with PF.

### 6.4. Energy Consumption Evaluation

In this section, we aim to validate whether the schemes that use the iBeacon receiver with low leakage power capability can provide more energy-efficient localization compared with the different methods using the WiFi module. To observe the energy consumption of our localization system, the power monitor of Monsoon Solution [42] was connected to the smartphone that runs at 3.96 V. For the analysis of the energy consumption according to the use of IMU sensors (accelerometer and gyroscope) and radio modules (WiFi and Bluetooth device) in our positioning system, our experiments were carried out using several operational modes: IMU sensors (IMU), IMU sensors and WiFi (IMU+WiFi); IMU sensors and Bluetooth (IMU+BT); and IMU sensors, Bluetooth, and WiFi (IMU+BT+WiFi), which correspond to the positioning methods P, PU1, PU2, and PU3, respectively.

**Figure 16:**
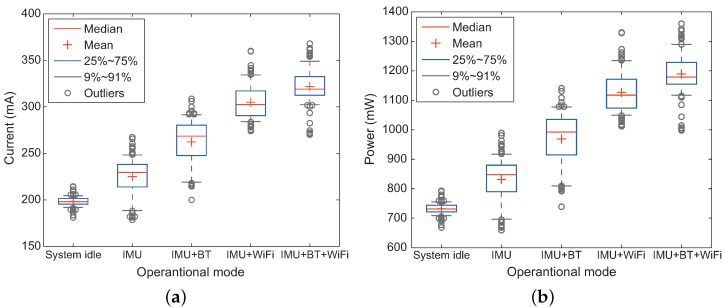
Statistics of the measured (**a**) current and (**b**) power in various experiment settings.

Figure 16 represents the boxplots of the measured current and power from the different operational modes. In this figure, we can observe that modes using RF modules consume more energy compared to that using only IMU sensors, becoming particularly remarkable in the experiments using WiFi module, such as IMU+WiFi and IMU+BT+WiFi. By comparing between IMU+BT and IMU+WiFi, it is also observed that the average current and power for the WiFi module are noticeably higher than those for the Bluetooth device. This is because the Bluetooth device on the smartphone employed in our experiments is based on the low energy technology called BLE (also known as Bluetooth 4.0). Thus, using IMU+BT (i.e., PU2), we can achieve positioning performance with high accuracy and energy efficiency.

## 7. Conclusions

As a solution to the problem of indoor pedestrian positioning that suffers from substantial errors and large bias, we have presented an indoor localization system using simple dead reckoning (DR) method, fingerprinting approach using machine learning and energy-efficient iBeacon, and SKPF algorithm, the enhanced KF proposed in this paper. Using the DR method, the position of the user is predicted by the sensory data (acceleration and heading) of the mobile phone. Instead of GPS, the positional measurement of the user can be obtained from the fingerprinting approach in our positioning method. However, there are still errors in positional information obtained from both the DR and fingerprinting method.

The core of our localization system is the SKPF algorithm that improves KF by leveraging the unscented transformation of UKF and the weighting method of PF. The SKPF algorithm can achieve better positioning performance than KF and UKF and competitive performance compared to PF, and it can provide higher computational efficiency compared with PF. The SKPF algorithm in our localization system is used to provide enhanced positioning accuracy by integrating noisy positional information estimated by DR method and the location data obtained by the fingerprinting approach with uncertainty. We aim to design the localization scheme that can realize the high positioning accuracy, computational efficiency, and energy efficiency through the SKPF and iBeacon indoors. Empirical results in a building show that the SKPF algorithm in our indoor localization system can provide very satisfactory performance in aspect of positioning accuracy and computational cost compared with KF, UKF, and PF. It is also shown in the test results that the positioning system using iBeacon signal as a location feature for the fingerprinting method can achieve more energy-efficient localization than using WiFi signal. Our future research is to apply our localization system to very different scenarios such as 3D indoor environments, along with more tests for the validation for the system.

References

## Figures and Tables

**Table 1:** Positioning Methods for the Experimental Tests.

Notation	Description
P	Prediction using acceleration and heading
PU1	P and update using measurement from “WiFi RSS+Heading”
PU2	P and update using measurement from “iBeacon RSS+Heading”
PU3	P and update using measurement from “WiFi/iBeacon RSS+Heading”
